# Prevalence and associated factors of suicidal behavior among pregnant mothers in southern Ethiopia: a cross-sectional study

**DOI:** 10.1186/s12889-022-12957-9

**Published:** 2022-03-12

**Authors:** Alemayehu Molla, Jemberu Nigussie, Bekahegn Girma

**Affiliations:** 1 Department of Psychiatry, College of Health and Medical Science, Dilla University, Dilla, Ethiopia; 2 Departments of Nursing, College of Health and Medical Sciences, Dilla University, Dilla, Ethiopia

**Keywords:** Antenatal care, Ethiopia, Suicidal behavior, Mothers

## Abstract

**Background:**

Suicidal behavior among pregnant mothers is one of the most common psychiatric emergencies that require a major public health concern by researchers and mental health task forces. Pregnant mothers experience suicidal attempt, which is a fatal problem to end life. Therefore, there was a need to assess the prevalence and associated factors of suicidal behavior among pregnant mothers to integrate mental health care, particularly suicide, with maternal management.

**Methods:**

A cross-sectional study was conducted among 504 pregnant mothers in the Gedeo zone, southern Ethiopia. Suicidal behavior was assessed using revised suicidal behavior questionnaire (SBQ-R) with a total score of 3-18; those scoring ≥7 were considered as having Suicidal behavior. Data were entered into Epi-data 3.1 and analyzed using SPSS version 20. Bivariate and multivariate binary logistic regression analysis was performed to identify associated factors of suicidal behavior. Variables with a *P*-value less than 0.05 with 95% CI were considered statistically significant.

**Results:**

In this study, the overall prevalence of suicidal behavior among pregnant mothers was 47(9.3%) with 95% CI (7.1- 11.9). Regarding the factors; being unmarried [AOR = 5.69, 95% CI, (1.19, 27.23)], gestation age greater than 27 weeks, [AOR = 4.92, 95% CI (1.67, 14.53)], history of having chronic medical illness [AOR = 4.47, 95% CI (1.35, 14.85)], depression [AOR = 4.20, 95% CI (1.90, 9.28], and intimate partner violence [AOR = 7.60, 95% CI (3.27, 17.67)] were significantly associated with suicidal behavior at *P* value less than 0.05 and corresponding 95% CI.

**Conclusion:**

Pregnant mothers in the community had a high prevalence of suicidal behavior compared to studies conducted among general populations. It is better to include and implement the assessment of suicidal risk factors as a primary treatment package for pregnant mothers, training of health extension workers and other primary health workers on how to assess the risk of suicide among pregnant mothers is warranted.

## Background

Suicidal behavior is a complex process that includes suicidal ideation, planning, attempting suicide, and the final act of committing suicide [1]. It is more specifically classified into three categories: suicidal ideation, a suicide plan, and suicide attempt [1, 2]. Suicide remains a significant social and public health problem. World Health Organization (WHO) reported that more than 1 million people died by suicide worldwide each year [1, 3]. Suicide is increasingly becoming a notable global public health problem and is now the tenth leading cause of death in the general population [4]. An estimated 75.5% of suicides occur in low- and middle-income countries (LMICs) [5]. In the general population, the annual global suicide rate is 11.4 per 100, 000 population or one death every 40 s [6].

Different studies showed that suicide is one of the leading causes of maternal mortality during pregnancy [7–12] and there is increment of suicidal ideation and behavior (SIB) among pregnant mothers from time to time [13]. Suicidal ideation and suicide attempt during pregnancy are associated with numerous consequences that adversely affected maternal and infant outcomes, like psychiatric disorders, fetal growth restriction, premature labor, cesarean delivery, respiratory distress, depression, and addicted alcohol [12, 14–16]. An emerging body of evidence showed that suicidal ideation is a precursor and predictor of later suicide attempts and completions [17]. It is a devastating event and one of the leading causes of maternal deaths during the period of pregnancy [18, 19]. Suicide was found to be the fifth most common cause of death during pregnancy and suicidal behavior occurs in 3 to 14% of the obstetric population [20, 21].

The move from pregnancy to motherhood can adversely influence women’s mental well-being [22, 23]. Depression and anxiety are also common problems during and after pregnancy, which intern increases suicidal behavior [24–26]. Pregnant mother with depression and anxiety are more likely to experience thoughts of death and engage in suicidal behavior compared with other pregnant mothers [27]. As a consequence, suicidal behavior is not only a problem for victims, but also had effect on emotional disturbance of the family members [28, 29]. The reported prevalence of suicidal ideation during pregnancy varied widely in studies, ranging from 3 to 33% [30].

In Ethiopian culture, suicidal behavior is considered as a sin among family members with a potential risk of rejection. Although pregnant mothers are at high risk, there are limited studies on the magnitude of the problem among mothers in Ethiopia and there is no community based evidence regarding the problem. Therefore, this study is aimed to assess the prevalence and associated factors of suicidal behavior among pregnant mothers from the community in the Gedeo zone, Southern Ethiopia. Early detection of pregnant women with non-fatal suicidal thoughts and behavior is an important opportunity to direct suicide prevention efforts to those at high risk for suicide and therefore can help to prevent maternal mortality [7, 8, 31].

## Methods

### Study design and settings

A cross-sectional study was conducted among rural community residents of Gedeo zone in southern Ethiopia from February 1/2021 to 30/2021. Gedeo zone is one of the 11 zones found in the southern nations nationalities peoples region (SNNPR) with the administrative center of Dilla town located 360 km from Addis Ababa (the capital of Ethiopia) and a total of 276 health facilities were found in the zone [9, 10]. According to the 2007 census report, the total population of the zone was 1, 086,768 with 532,516 (49%) male and 554, 225 (51%) female [11]. A total of 179,677 households were counted in this Zone, which results an average 4.72 of persons to a household.

### Study subjects

All adult mothers with age ≥ 18 years and available during the data collection period were study participants. Mothers who are severely ill and have communication problems were excluded from the study.

### Sampling size and sampling procedure

The required sample size was determined using the single population proportion formula, considering the assumptions: standard normal distribution (Z = 1.96), confidence interval 95% and ⍺ = 0.05, *P* = 20.4% taken from a similar study carried out in Egypt [12], a margin of error (d) =5%. After using design effect 2 and a nonresponse rate of 5%, the final sample size was 525. The multistage sampling technique was used; first out of 8 districts in zone 3 districts were randomly taken by the lottery method and followed by random selections of nine sub-districts (3 in each). The proportional allocation was done based on the numbers of participants in each sub-district. The lists of pregnant mothers were obtained from health extension workers. The samples were selected proportionally from each sub-district after calculating the sampling interval (K) value for each. The starting point of the interval was identified by using lottery method. In case of more than one eligible pregnant mother in the household, the lottery method was used to select only one. For the eligible participants who were not found at home, the interviewers re-visited the home other times.

### Data collection instruments

The study questionnaire had socio-demographic characteristics, clinical, pregnancy, substance, psychosocial and suicidal-related factors. Social support was collected using Oslo-3 item social support scale which is commonly used to assess social support and has been used in several studies, the sum score scale ranges from 3 to 14, which has 3 categories: poor support 3-8, moderate support 9-11, and strong support 12-14 [14].

Symptoms of maternal depression were assessed using the Edinburgh Postnatal Depression Scale (EPDS) [15]. It consists of 10 item questions which examines the emotional state that occurred within the past 7 days. Each question has four possible answers with an interval of 0-3 and the overall score is out of 30. The probable cases were considered if the score is 13 and above, the tool 86% sensitivity and 78% specificity respectively [15].

Intimate partner violence was considered if participants expresses physical violence, sexual violence, and psychological aggression by intimate partner [16, 17]. Internal consistency (Cronbach’s alpha) of intimate partner violence in this study was 0.74.

The history of substance use was evaluated by yes or no questionnaires. The tool was adopted from the WHO ASSIST (Alcohol, Smoking, and Substance Involvement Screening Test). ASSIST is a valid screening test for identifying substance use in individuals and have varying degrees of substance use. Similarly, physical and mental illnesses were assessed by yes or no response questionnaires. The outcome variable was assessed using the revised suicidal behavior questionnaire. Reliability and validity tests among general population in prison were 79 and 95%, respectively. The most useful cut off score for community participants was 7; Cronbach’s alpha α = 0.79 and the tool has 93% sensitivity and 95% specificity [32].

### Data quality control

The instruments were initially prepared in English and then back translated into the local language to check consistencies. Pretest was carried out on 5% of the total sample size before the actual data collection period; training was given for data collectors and supervisors. The collected data were checked for completeness and consistency before data entry.

### Data analysis

The coded data were entered into Epi-data 3.1 and analyzed using SPSS version 20. Descriptive data were presented using frequencies and percentages within tables and texts. Binary logistic regression analysis was performed to determine each of the explanatory variables and variables with a *p*-value less than 0.2 during bivariate analysis were candidates for multivariate analysis [23, 33]. Multivariate binary logistic regression analysis was performed to determine the presence of significant association between independent variables and outcome variables. Finally, variables with *P*-values less than 0.05 were considered a statistically significant and the strength of the association was presented by an adjusted odds ratio with the corresponding 95% CI.

## Results

### Socio-demographic characteristics of the respondents

A total of 504 participants with a response rate of 96% were included in this study. The mean age (±SD) of the respondents was 27.56(±5.6). Among the respondents, the majorities were in the age range of 18 to 28 years 290(57.5%). Of the total participants, 381 (75.6%) were protestant in religion and 378(75%) were Gedeo in their ethnicity. The majority of the participants were married 488 (96.5%). The educational status of the participants indicated that 196 (39.3%) of them cannot read and write, but 167(33.1%) of their husbands can read and write. Regarding occupation, 315 (62.5% of the participants reported being a housewife. The majority, 428 (84.3%) of respondents lived in rural areas (Table 1).

**Table 1:** Distribution of participants by socio-demographic factors among pregnant mothers in the Gedeo zone, Ethiopia, 2020/21 (*n* = 504)

Variable	Frequency (*N* = 504)	Percent (%)
Age		
18-28 years	290	57.5
29-39 years	204	40.5
40-50 years	10	2
Religion		
Protestant	381	75.6
Orthodox	101	20
Others^a^	22	4.4
Marital status		
Married	488	96.8
Unmarried	16	3.2
Ethnicity		
Gedeo	378	75.0
Others^b^	126	24.8
Education status		
Can’t read and write	198	39.3
Can read and write	195	38.7
Primary	48	9.5
Secondary	41	8.1
College and above	22	4.4
Occupational status		
Government employed	39	7.7
Farmer	11	2.2
Private	59	11.7
House wife	315	62.5
Merchant	76	15.1
Others ^c^	4	0.8
Education status of husband		
Can’t read and write	114	22.6
Can read and write	167	33.1
Primary	110	21.8
Secondary	68	13.5
College and above	45	8.8
Residency		
Rural	425	84.3
Urban	79	15.7
Average monthly income		
< 1539 ETB	453	89.9
≥ 1539ETB	51	10.1

Others ^a^ = Muslim, Joba witness &no religion, ^b^ = Oromo, Amhara, Gurage, Sidema, Wolyita & silte ^c^ = no work& students

### Obstetric and clinical related factors

Regarding the obstetric-related factors of respondents, for about 237 (47%) of the participants, the gestational age of the current pregnancy ranges 14-27 weeks and of the total participants, 250 (49.6%) had more than three previous pregnancies. About 85(16.9%) of the respondents had an abortion history. Regarding current pregnancy, 452 (89.7%) of participants reported that their current pregnancy was planned. About 40 (7.9%) of the respondents reported a family history of mental illness, and only 16 (3.2%) of participants had a history of mental illness in themselves. Regarding the history of other chronic diseases, about 26 (5.2%) of participants had chronic illnesses, of these 19 (73.1%) had tuberculosis as a chronic disease (Table 2).

**Table 2:** Description of obstetric and clinical-related factors among pregnant mothers, in Gedeo zone, Ethiopia, 2020/21

Variables	Frequency	Percent (%)
Weeks of pregnancy		
< 14 weeks	110	21.8
14-27 weeks	237	47
> 27 weeks	157	31.2
Number of pregnancy		
Once	136	30
Twice	118	23.4
Three and more	250	49.6
Abortion history		
Yes	85	16.9
No	419	83.1
Number of abortion		
Once	74	97.1
Two times	11	12.9
Planed pregnancy		
Yes	452	89.7
No	52	10.3
Family history of mental illness		
Yes	40	7.9
No	464	92.1
History of mental illness		
Yes	16	3.2
No	488	96.8
History of chronic illness		
Yes	26	5.2
No	469	93
Types of chronic illness		
Tuberculosis	19	73.1
HIV	4	15.4
Hypertension	3	11.5

### Psychosocial and substance-related factors

Among participants, more than half 294 (58.3%) of respondents had moderate social support and 60 (11.9%) of them reported intimate gender-based violence. Regarding the assessment done on depression using the Edinburg depression scale, approximately 87 (17.3%) of the respondents had depression and 79 (15.7%) of the participants had a history of alcohol use in their life (Table 3).

**Table 3:** Distribution of psychosocial and substance-related factors among pregnant mothers, in Gedeo zone, Ethiopia, 2020/21 (*n* = 504)

variables	Frequency	Percent (%)
Social support		
Poor	136	27
Moderate	294	58.3
Strong	74	14.7
Intimate partner Violence		
Yes	60	11.9
No	444	88.1
Depression		
Yes	87	17.3
No	417	82.7
Life time use of alcohol		
Yes	79	15.7
No	425	84.3
Life time use of khat		
Yes	2	0.4
No	502	99.6
Current use of alcohol		
Yes	59	11.7
No	445	88.3
Current use of khat		
Yes	1	0.2
No	503	99.8

### Prevalence of suicidal behavior among pregnant mothers

In this study, the overall prevalence of suicidal behavior among pregnant mothers was 9.3% (47) (with 95% CI (7.1-11.9). Of the participants with suicidal behavior, 25 (53.2%) had suicidal ideation, 17 (36.2%) had plans, and 5 (10.47%) attempted suicide.

### Factors associated with suicidal behavior among pregnant mothers

In multivariate binary logistic regression; being unmarried, gestational age greater than 27 weeks, chronic medical illness, depression, and intimate partner violence were statistically significant with suicidal behavior at a *p*-value less than 0.05.

The odds of having suicidal behavior among unmarried respondents were 5.69 times higher compared to married respondents [AOR = 5.69, 95% CI (1.19, 27.23)]. In this study, participants who have gestational age greater than 27 weeks were 4.92 times more likely to have suicidal behavior compared to respondents who have gestational age less than 14 weeks [AOR = 4.92; 95% CI (1.67, 14.53)].

Participants with a history of chronic medical disease were 4.47 times more likely to report suicidal behavior compared to their counterparts [AOR = 4.47; 95% CI (1.35, 14.85)]. The odds of having suicidal behavior among respondents with depression were 2.32 times higher compared to participants without depression [AOR = 4.20; 95% CI (1.90, 9.28)].

Intimate partner violence was also another factor associated with suicidal behavior. Participants with a history of intimate partner violence were 7.60 times more likely to have suicidal behavior compared to their opposite groups [AOR = 7.60; 95% CI (3.27, 17.67)] (Table 4).

**Table 4:** Bivariate and multivariate logistic regression analysis of the factors associated with suicidal behavior among pregnant mothers in Gedeo zone, Ethiopia, 2020 / 21 (*N* = 504)

Explanatory variables	Suicidal behavior		COR (95%CI)	AOR (95%CI)	*p*-value
	Yes	No			
Marital status					
Currently married	39	449	1	1	
Currently unmarried	8	8	11.51(3.55,37.39)	5.69 (1.19, 27.23)	0.03
Week of pregnancy					
Less than 14 weeks	8	102	1	1	
14-27 weeks	17	220	0.99 (0.41, 2.36)	2.11(0.70, 6.39)	
Greater than 27 weeks	22	135	2.08 (0.89, 4.86)	4.92(1.67, 14.53)	0.004
Planed pregnancy					
Yes	39	413	1	1	
No	8	43	1.97 (0.87, 4.49)	1.84(0.58, 5.58)	
Family mental illness					
Yes	12	28	5.25 (2.46, 11.22)	2.71(0.98, 7.46)	
No	35	429	1	1	
History of mental illness					
Yes	3	13	2.33 (.64, 8.49)	0.40(0.06, 2.69)	
No	44	444	1	1	
Medical illness					
Yes	8	18	4.98 (2.04,12.19)	4.47 (1.35,14.85)	0.014
No	39	439	1	1	
Depression					
Yes	22	65	5.31 (2.96, 10.47)	4.20 (1.90,9.28)	0.001
No	25	392	1	1	
Social support					
Poor	21	115	2.52 (0.91, 6.99)	2.35 (0.71,7.77)	
Moderate	21	273	1.06 (0.39, 2.92)	1.04 (0.33, 3.30)	
Good	5	69	1	1	
Intimate partner violence					
Yes	21	39	8.66 (4.67, 16.78)	7.60 (3.27,17.67)	0.001
No	26	418	1	1	

## Discussion

Even though pregnant mothers are more prone to suicide, to our knowledge there is no community-based evidence regarding the problem in our country. Therefore this study is intended to fill the gap by assessing the prevalence and associated factors of suicidal behavior among pregnant mothers and show scope of the problem in the study areas. The findings of this study will help the institution to develop appropriate plans and intervention to mothers with suicidal risk. Furthermore, the result of this study will provide information for policymakers to design an appropriate plan for the problem and to the Ministry of health to promote the integration of psychiatric services in the treatment of pregnant mothers.

The current study showed that about 9.3% with 95% CI (7.1-11.9) of pregnant mothers had suicidal behavior in communities. The finding of this study was similar to an institutional-based study conducted in Hawassa, Ethiopia in which 11.8% of pregnant mothers had suicidal ideation [34].

Regarding prevalence, the finding was lower than other studies conducted in South Africa 18% [35] & 27.5% [36], an institutional study conducted among postnatal mothers in Northwest Ethiopia 14% [37], another institutional study conducted in Jimma, Ethiopia 13.3% [38], Egypt 20.4% [12] and Brazil 13.3% [21].

However, the finding was higher than the study conducted in the United Kingdom 2% [39], Tanzania 0.8% [40], and the community-based study conducted in Ethiopia among general population 4% [41]. The possible reason for this variation might be the difference in the study design in which a large study (follow-up) was used in the study conducted in United Kingdom [39]. The study conducted in South Africa [21] used a pilot study among pregnant mothers in hospitals using a small sample size. The setting of the study could be other possible reasons for this variation, in which high-risk mothers from the obstetric treatment unit in hospitals were included for the study conducted in South Africa [35] in which mothers may have a high suicidal behavior due to additional complications and hospital-related factors, but our study was carried out in the community among healthy pregnant mothers. The high prevalence of suicidal behavior among previous studies conducted in Ethiopia might be due to the risk of having complications or other related medical problems since all of the previous studies in Ethiopia were institutional-based studies [37, 38].

Furthermore, the difference in suicidal behavior assessment tools could be another possible reason for this discrepancy in the prevalence of suicidal behavior among pregnant mothers, the study conducted in Brazil used the suicidal part of Mini International Neuropsychiatric Interview (MINI) which included nine questions and of this suicidal behavior was evaluated by three questions, but our study used the revised SBR with a likert scale and cut off point 7. Regarding tool difference, the study conducted in Egypt used the Beck suicidal scale, which is the clinical scale of choice, focusing on the cases seen in the clinical settings [12]. Sociocultural, sample size and timing of study difference could also be another possible reason for the difference in the prevalence of suicidal behavior among pregnant mothers. Regarding the cultural effect of reporting suicidal behavior, most communities in developing countries including Ethiopia, consider reporting suicide is not culturally acceptable and stigmatizing issue, so this could be a possible reason for the low prevalence of suicidal behavior in our study compared to studies done in other countries.

Regarding factors, depression was significantly associated with suicidal behavior and this was supported by studies conducted in Brazil [21], South Africa [35], United Kingdom [39], Egypt [12], an epidemiological review [30], and another study in South Africa [36]. Many of these previous studies reported that comorbid depression is highly associated with suicidal behavior. The possible reason might be that depression decreases monoamine neurotransmitters like serotonin, in which studies have shown an association between decreased neurotransmitter level and suicidal behavior [42]. It may also be due to the direct effect of depression that makes patients feel helpless, isolated, and worthless. Another factor associated with suicidal behavior was the presence of intimate partner violence. This finding was supported by studies conducted in Egypt [12], Brazil [21], South Africa [35], an epidemiological review conducted in developed countries and study conducted in American countries [43]. It is known that the psychosocial support of husband is vital for the health and well-being of pregnant mothers [44]. But, if mothers experience verbal and physical violence from their partner, they lose hope and plan. Due to the above and related reasons, pregnant mothers might have suicidal behavior compared with the mother having good support from their partner [45].

Suicidal behavior was also associated with the pregnant mothers with history of chronic medical illness and this was in agreement with studies conducted in South Africa [36], and a hospital-based study conducted in Northwest Ethiopia [37]. The possible reason for this might be due to the double burden of chronic disease and pregnancy [46, 47]. Lifetime use of some medications used for chronic medical illness; like HIV might be risk factors for suicide [48].

According to current findings, pregnant mothers with a gestational age greater than 27 weeks and unmarried mothers were also at high risk of suicidal behavior. The possible explanation might be that most mothers with single marital status lack physical and psychological support, making them feel hopeless and helpless for all life activities [49]. The finding regarding gestational age was not consistent with studies conducted in Sweden [50, 51]. The possible reason for suicidal risk among pregnant mothers with gestational age greater than 27 weeks in the current study might be due to antenatal depression which is risk for suicide and common in second and third trimesters as reported from previous systematic review and meta-analysis study and other single study conducted on depression and pregnancy in Ethiopia [52, 53].

### Limitations of the study

First, since data collection was an interview method, it might face the recalling problem of some symptoms. Second, since it is a cross-sectional study design, it cannot allow establishing a temporal relationship between outcome variables and significant associated factors.

## Conclusions

The current study showed that pregnant mothers in the community had a high prevalence of suicidal behavior compared to studies carried out among general populations. Mothers; with depression, intimate partner violence, comorbid medical illness, and gestational age of more than 27 weeks and unmarried were high risks for suicidal behavior. It is better to include and implement assessment of suicidal risk factors as a primary treatment package for pregnant mothers, training of the health extension workers and other primary health workers on how to assess risk of the suicide among pregnant mothers is warranted.

## Data Availability

All raw data included in the manuscript can be accessed from the corresponding author through the email address of ‘alexmolla09@gmail.com’ with reasonable request.

## Abbreviations

AOR: Adjusted Odds Ratio
CI: Confidence Interval;
CID: Composite International Diagnostic Interview
COR: Crude Odds Ratio
EPDS: Edinburgh Postnatal Depression Scale
IPV: Intimate Partner Violence
MINI: Mini International Neuropsychiatric Interview
PLWE: People Living with Epilepsy
PHQ-9: Patient Health Questionnaire
SBQR: Suicide Behaviors Questionnaire-Revised
SNNRE: Southern Nation Nationalities and Regions of Ethiopia
